# Refinement of primate **copy number variation **hotspots identifies candidate genomic regions evolving under positive selection

**DOI:** 10.1186/gb-2011-12-5-r52

**Published:** 2011-05-31

**Authors:** Omer Gokcumen, Paul L Babb, Rebecca C Iskow, Qihui Zhu, Xinghua Shi, Ryan E Mills, Iuliana Ionita-Laza, Eric J Vallender, Andrew G Clark, Welkin E Johnson, Charles Lee

**Affiliations:** 1 Department of Pathology, Brigham and Women's Hospital, 75 Francis Street, Boston, MA 02115, USA; 2 Harvard Medical School, 25 Shattuck Street, Boston, MA 02115, USA; 3 Department of Anthropology, University of Pennsylvania, 3260 South Street, Philadelphia, PA 19104, USA; 4 Mailman School of Public Health, Columbia University, 722 West 168th Street, New York, NY 10032, USA; 5 New England Primate Research Center, One Pine Hill Drive, Southborough, MA 01772, USA; 6 Department of Molecular Biology and Genetics, Cornell University, 107 Biotechnology Building, Ithaca, NY 14853, USA

## Abstract

**Background:**

Copy number variants (CNVs), defined as losses and gains of segments of genomic DNA, are a major source of genomic variation.

**Results:**

In this study, we identified over 2,000 human CNVs that overlap with orthologous chimpanzee or orthologous macaque CNVs. Of these, 170 CNVs overlap with both chimpanzee and macaque CNVs, and these were collapsed into 34 hotspot regions of CNV formation. Many of these hotspot regions of CNV formation are functionally relevant, with a bias toward genes involved in immune function, some of which were previously shown to evolve under balancing selection in humans. The genes in these primate CNV formation hotspots have significant differential expression levels between species and show evidence for positive selection, indicating that they have evolved under species-specific, directional selection.

**Conclusions:**

These hotspots of primate CNV formation provide a novel perspective on divergence and selective pressures acting on these genomic regions.

## Background

Copy number variants (CNVs) are gains or losses of genomic material, and are now known to constitute a major source of genetic polymorphism in humans [1]. High resolution studies of human CNVs permit the investigation of the mechanisms causing CNV genesis [2-5], the potential impact of CNVs on gene expression [6], the contribution of CNVs to phenotypic variation [7], and the role that CNVs have in disease manifestation and mitigation [8-10].

CNV hotspots are highly plastic genomic regions where mutations leading to copy number differences between individuals occur more frequently than expected [11,12]. Among non-human primates, CNV maps have been developed for the chimpanzee (*Pan troglodytes*) [11,13] and rhesus macaque (*Macaca mulatta)*[14]. We have constructed a second-generation, high-resolution CNV map for the rhesus macaque and combined it with similar CNV data for the chimpanzee and ultra-high-resolution CNV data from humans to determine comprehensively the location and structure of primate CNV hotspots. These genomic regions appear to have an elevated likelihood of positive selection, based on nucleotide level conservation and transcriptional data.

## Results

### Generating a high-resolution rhesus macaque CNV map

In order to identify primate hotspots for CNV formation, we compiled CNV datasets for human, chimpanzee and rhesus macaque. We used two recently published CNV discovery studies in humans [1,15] to assemble a non-redundant dataset of 12,146 human CNVs that are larger than 437 bp in size (Table S1 in Additional file 1). The chimpanzee dataset was composed of 438 merged CNV regions from the most comprehensive study documenting within-chimpanzee copy number variation [13]. For generating a comparable rhesus macaque CNV dataset, we designed a rhesus macaque-specific array comparative genomic hybridization (aCGH) platform containing 950,843 unique 60-mer oligonucleotide probes.

**Figure 1:**
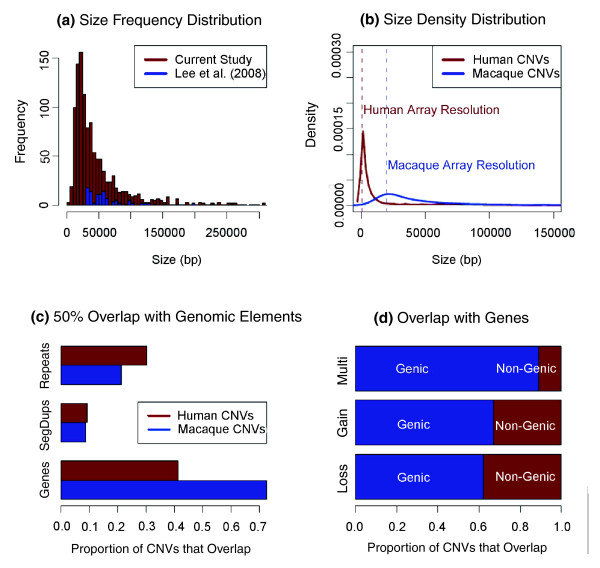
**Comparison between human and rhesus macaque CNVs and enrichment of genic CNVs in rhesus macaques**. **(a) **Size distribution and summary statistics of the observed CNVs. The frequency distribution of CNV sizes. CNVs that are larger than 300 kb were omitted from the graph. The red and blue bins show the frequency distribution of the CNV sizes observed in this study and in Lee *et al. *[14], respectively. Note the overall increase in the frequency of detected CNVs in this study. **(b) **The size distributions of human and macaque CNVs are similar (that is, as the frequency increases, the size gets smaller). However, the human CNVs were identified with a higher resolution platform and so there are smaller human CNVs detected compared to rhesus macaque. **(c) **The overlap with genomic components, such as repeats and segmental duplications, are similar for human and macaque CNVs. However, rhesus macaque CNVs are much more likely than human CNVs to overlap with genes. **(d) **As observed in humans, most of the rhesus macaque CNVs that overlap with genes are gains or multi-allelic CNVs. Note the dramatic increase in the ratio of genic CNVs.

This custom aCGH platform was applied to the genomes of 17 unrelated macaques, resulting in the identification of 1,160 CNVs (Table S2 in Additional file 1). To identify CNVs, we required a minimum of five consecutive probes with log_2 _ratios significantly deviating from 0 (Figure S1 in Additional file 2), and conducted extensive supporting analyses (Supporting methods in Additional file 3 and Figure S2 in Additional file 2). This approach provided an approximate 15 kb effective resolution to identify CNVs across the rhesus macaque genomes, as our probes are distributed with an average spacing of less than 3 kb (Figure S3 in Additional file 2). Approximately 95% (1,096) of the CNVs reported in this study have not been documented previously (Figure S4a,b in Additional file 2), and roughly half of these are losses of genetic material compared to the reference individual (Figure S4c in Additional file 2). More than 60% (698) of these CNVs are smaller than the effective resolution (approximately 40 kb) of an earlier study [14] (Figure 1a). We modeled the frequency distribution of the macaque CNVs (Figure S5 in Additional file 2) and estimated that thousands of common macaque CNVs are yet to be identified (Supporting methods in Additional file 3 and Table S3 in Additional file 1).

The size distribution of the rhesus macaque CNVs identified in this study is similar to that reported for human CNVs, in that the number of smaller CNVs increases exponentially. However, the large differences in resolution between these studies create substantial disparity in the size distribution of known human and rhesus macaque CNVs (Figure 1b). Rhesus macaque CNVs overlap with annotated sequences, such as segmental duplications and repeats, in proportions comparable to CNVs in humans (Figure 1c). In contrast to human CNVs, 843 (approximately 74%) of the macaque CNVs overlap with *Ensembl *gene predictions [16] (*P *< 0.001, Kolmogorov-Smirnov test; Figure S6 in Additional file 2). This is concordant with the study by Lee *et al. *[14], which showed that 68 of the 124 (55%) rhesus macaque CNVs identified are genic. In particular, we found that almost 90% (82 of 92) of the multiallelic rhesus macaque CNVs overlap with genes (Figure 1d).

### Human CNVs overlap with non-human primate CNVs more than expected by chance alone

**Figure 2:**
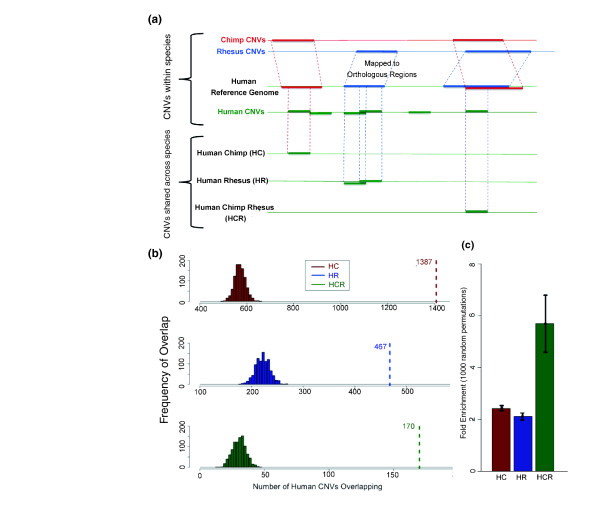
**Enrichment analyses of primate CNV hotspots**. **(a) **Chimpanzee and rhesus macaque CNV coordinates were converted to human reference build hg18 coordinates using the Galaxy Liftover tool. HC and HR CNVs are human CNVs that overlap one or more chimpanzee or macaque CNV(s), respectively, when using a 50% overlap criteria. For human CNVs that overlap both chimpanzee and macaque CNVs (HCR), critera were employed that required at least a 20% overlap between CNVs from any two species and a minimum of 50% overlap for overlapping CNVs from all three species. **(b) **We generated 1,000 random permutated CNV datasets containing CNVs of similar size distribution to the 12,146 human CNVs identified in Park *et al. *[15] and Conrad *et al. *[1]. The CNVs in each permuted human dataset were then assessed for their overlap with known chimpanzee and rhesus macaque CNVs. The *x*-axis represents the number of human permuted CNVs that overlap with chimpanzee CNVs (red distribution) or macaque CNVs (blue distribution) during these permutation iterations. The green distribution represents human CNVs that overlap with both chimpanzee and rhesus macaque (HCR) CNVs during these same permutation iterations. The *y*-axis represents the frequency of the permutated human CNVs that fall into each category. The dotted vertical lines indicate the actual number of overlaps with either chimpanzee CNVs (1,387), rhesus macaque CNVs (467) or both (170). **(c) **Based on the expectation of overlap with 1,000 random sets of intervals depicted in (b), the fold enrichment of human CNVs that overlap with chimpanzee (HC) or rhesus macaque (HR) CNVs is plotted, as is the fold enrichment of human CNVs that overlap with both chimpanzee and macaque CNVs (HCR). Error bars represent 1 standard deviation from the mean fold enrichment.

We used the LiftOver tool (UCSC Genome Browser) to map the rhesus macaque CNVs onto the human genome reference sequence (hg18; Figure 2a). The chimpanzee CNVs were already reported with orthologous hg18 genome coordinates [13]. Next, we identified human CNVs that overlap with chimpanzee and macaque CNVs. Specifically, 1,387 distinct human CNVs overlapped with 556 chimpanzee CNVs (HC: human + chimpanzee), 467 human CNVs overlapped with 385 rhesus macaque CNVs (HR: human + rhesus) and 170 human CNVs overlapped with both chimpanzee and rhesus macaque CNVs (HCR: human + chimpanzee + rhesus) (Table S4 in Additional file 1).

To test whether the overlap of human CNVs with chimpanzee and rhesus macaque CNVs is more than expected, we simulated 1,000 CNV datasets that mimic the size distribution of the actual human CNVs. In this manner, we effectively eliminated any bias in size distribution due to differences in resolution of the different human and nonhuman primate CNV discovery projects. From these simulated datasets, we constructed expected distributions of HC, HR and HCR CNVs with existing chimpanzee and macaque CNV datasets (Figure 2b). We subsequently calculated the deviation of the actual values from the expected distribution and found that there was significant enrichment for HR, HC and HCR, with HCR being the most enriched (*P *< 0.01, Kolmogorov-Smirnov test; Figure 2c). Finally, we conducted a similar, reciprocal analysis to show that macaque CNVs overlap with human CNVs more than expected by chance (Figure S6 in Additional file 2), and that there is no particular size cluster driving this enrichment (Figure S7 in Additional file 2).

### Primate CNV hotspots overlap regions of recurrent human CNV formation

**Figure 3:**
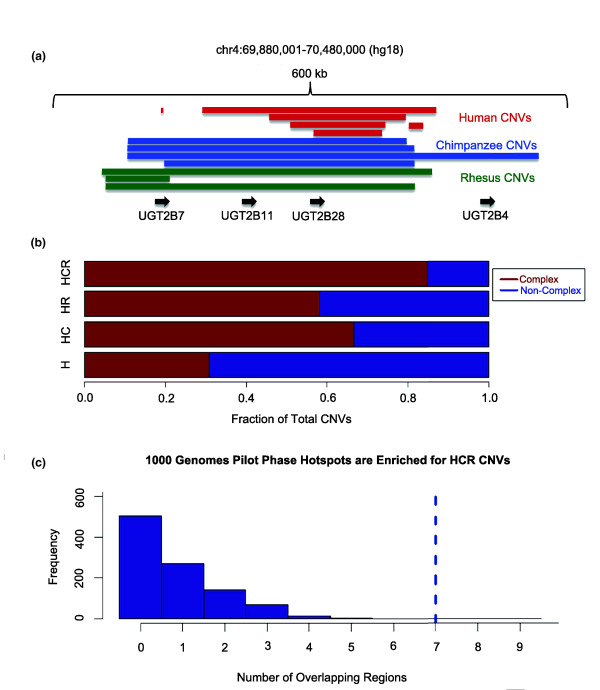
**Primate CNV hotspots are more likely to be complex in nature**. **(a) **Depiction of a typical primate CNV hotspot region, which is complex in nature, based on the presence of multiple overlapping CNVs with different breakpoints. Multiple members of a single gene family are present in this genomic region and may be contributing to some of the different CNV formations. CNVs were defined as complex based on whether they reciprocally overlapped other CNVs by less than 50% [1]. **(b) **The ratio of complex and non-complex CNVs in primate CNV hotspot and non-hotspot regions. Note the greater number of complex CNVs among HCR CNVs. **(c) **Enrichment analyses for the 1000 Genomes Project hotspots overlapping HCR CNVs. We iterated 1,000 intervals that mimic the size distribution of 55 human CNV hotspot regions [17], and constructed the expected overlap distribution of these intervals. Blue bars show the expected distribution and the dotted line marks the observed overlap. Here, we are showing that the observed overlap is much higher than expected by chance independent of the size of the intervals (*P *< 0.001, Kolmogorov-Smirnov test).

In HCR CNVs, we observed an increased frequency of 'complex' human CNVs (that is, the presence of multiple overlapping CNVs in the same genomic region). This observation is consistent with studies on the recurrence of CNV formation among different individuals [1] (Figure 3a). Of the 12,146 human CNVs used in this study, only 31.27% could be defined as complex CNVs. This proportion increases to 66.55% for HC, 58.03% for HR and 84.47% for HCR CNVs (*P *< 0.01 for all three categories, χ^2 ^with Yates correction; Figure 3b). To provide additional evidence regarding the genomic overlap of primate CNV formation hotspots and the location of human complex CNV regions, we used structural variation data analyses from the 1000 Genomes Project Pilot Phase data [17]. This study provided nucleotide-resolution breakpoints for most of the deletion CNVs that they identified, and identified 55 'human CNV hotspots' that contained at least a fivefold enrichment of CNV formation, using a 500 kb sliding window [17]. To accurately test whether primate hotspots overlap with these human CNV hotspots more often than expected, we generated 1,000 random intervals that mimic the size distribution of the human CNV hotspots to construct the expected distribution of recurrent overlap. Of the 55 human CNV hotspots identified, 23 (41.18%) overlapped with HC or HR CNVs in our study and 7 (12.73%) overlapped with HCR CNVs. These values indicate that primate hotspots of CNV formation overlap with regions of recurrent human CNV formation significantly more than expected (*P *< 0.01, fourfold enrichment, Kolmogorov-Smirnov test; Figure 3c).

### Only a small fraction of HCR CNVs evolve under neutral conditions

To quantify and understand the evolutionary mechanisms through which the primate CNV hotspots evolved and to delineate their possible functional impact, we collapsed the 170 HCR CNVs into a manually curated, non-redundant list of 34 primate CNV hotspot regions (Table S4 in Additional file 1). We found only four regions that do not overlap a gene, regulatory element, or disease-associated region. These four hotspot regions do not contain complex CNVs in humans (that is, each harbors CNVs with similar breakpoints) and are much smaller in size, with a mean length of approximately 2.7 kb, in contrast to a mean length of approximately 71 kb for the other CNV hotspot regions. Two of the four non-complex hotspot regions are overlapped almost entirely by transposable elements and repeat-rich DNA segments. The third hotspot region is entirely composed of a single segmental duplication and the fourth hotspot region resides in the repeat-rich subtelomeric region of chromosome 8.

The simplest explanation for the presence of a primate CNV hotspot is that it evolved under neutral conditions with little or no selective pressure acting on it. The first task is to distinguish between events that evolved under neutral conditions and non-neutral conditions. Because it is unlikely that the genomic plasticity itself is selected for or against, we suggest that the selection acts not on the elements that maintain genomic plasticity, but rather on the functional elements that reside within the CNVs. Hence, the selection for the genomic plasticity occurs indirectly and should be parallel to the selection acting on the functional content of the CNV. As such, if no significant selective pressure is acting on randomly generated genomic plasticity, we would expect to observe a depletion of functional loci, which by definition are under selection.

**Figure 4:**
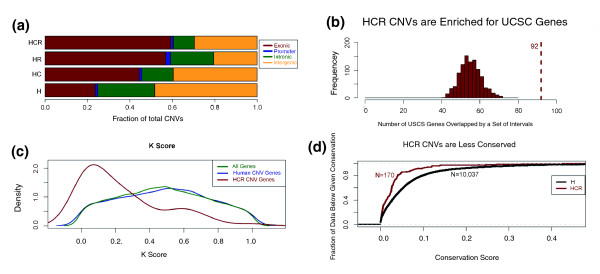
**Primate CNV hotspots overlap functional regions**. **(a) **CNVs were classified as intergenic, intronic, or exonic, based on whether any part of them overlapped with genes. Promoter regions were defined as the 2-kb region immediately upstream of the transcription start site of a gene. **(b) **Enrichment analysis for the HCR CNVs that overlap with exons of UCSC gene track. For calculating the significance independent of the size distribution, we generated 1,000 intervals that mimic the size distribution of the HCR CNVs and plotted the distribution of their genic overlap. The dotted red line indicates the number of observations, whereas the red-bins show the expected distribution (*P *< 0.001, Kolmogorov-Smirnov test). **(c) **The *K *values for all genes, genes that overlap with human CNVs, and genes that overlap with HCR CNVs. We plot here the density of *K *values, which is a measure of positive selection [18]. The genes that overlap with HCR CNVs have significantly lower *K *values (*P *< 0.001, Kolmogorov-Smirnov test), indicating that they are more likely to evolve under positive selection. **(d) **A cumulative fraction plot of conservation for primate hotspot and non-hotspot CNVs. Conservations scores were obtained using the phastCons on 17-species multiz track from the UCSC Genome Browser [31]. The D and P-values were calculated using a Kolmogorov-Smirnov test. Combined, these data indicate that most of the genes overlapping HCR CNVs are evolving under lineage-specific positive selection.

In our analysis, we found that, even with conservative measures, the vast majority of HCR CNVs fall into regions with functional relevance, in stark contrast with non-hotspot human CNVs (Figure 4a). To test whether our observations are statistically significant, we iterated two different sets of 1,000 intervals that mimic the size distribution of the HCR CNVs and the 34 curated hotspots regions respectively. We then used these datasets to build expected distributions of genic overlap of HCR CNVs and hotspot regions to calculate statistical significance. We found that the 170 HCR CNVs and 34 hotspots overlap with genes more than expected by chance and in a manner that is independent of their size distribution (*P *< 0.01, Kolmogorov-Smirnov test; Figure 4b; Figure S8a,b in Additional file 2). Therefore, our observations are consistent with the notion that most of the primate CNV formation hotspots did not evolve under relaxed (neutral) evolutionary pressure alone, and other evolutionary scenarios (such as balancing or positive selection) should also be considered.

### HCR CNVs evolve primarily under directional positive selection in humans

Most HCR CNVs are associated with genes, and primarily with gene families (Table S4 in Additional file 1). To quantify possible selection on these genes, we used the recently published dataset of positively selected genes in primates [18]. Specifically, this study examines the heterozygosity-within-species at a given locus and its surrounding area, while controlling for neutral mutation rate using the cross-species divergence, in order to calculate an empirical measure of positive selection, *K *(0 ≤ *K *≤ 1), within a species. Loci with lower κ values are more likely to have evolved under positive selection. Using this measure, we found that approximately 36% of the genes located within HCR CNVs are positively selected, as opposed to only 9% of the genes that overlap with H CNVs (*P *< 0.01, χ^2 ^with Yates correction; Figure 4c; Figure S8c in Additional file 2). We further observed that *K *values for the genes that overlap with HCR CNVs are significantly lower than those that overlap with H CNVs (*P *< 0.01, Kolmogorov-Smirnov test).

In addition, we found that at the nucleotide level, HCR CNVs are much less conserved than CNVs found only in humans (Figure 4d; Figure S8d in Additional file 2). Given that most of the HCR CNVs overlap with functional sequences, one explanation for the sequence divergence between species could be that these genomic regions are evolving under species-specific selective pressures. The sequence divergence could subsequently lead to different transcripts or differences in expression levels, as a result of changes in gene regulatory elements. Because several well-studied immune gene families (for example, beta defensins, *HLA, PCDHB *and *LILR *families) are indeed riddled with HCR CNVs and are known to evolve under balancing selection [19,20], it is possible that balancing selection has prevented the copy number at these loci from being fixed within the three different species.

## Discussion

In this study, we identified 170 human CNVs located within 34 primate hotspot regions of CNV formation. The structurally plastic hotspots appear to have remained active in the three lineages despite being separated by over 25 million years of evolution. The majority of primate hotspots overlap with functional genomic elements, especially genes related to immunity. A significant portion of these genes that overlap primate hotspots appear to have evolved under positive selection (Figure 4c) and some of these genes are also known to be evolving under balancing selection in humans (for example, the *HLA*, *PHDB*, and *LILR *families). As such, the evolution and maintenance of primate CNV hotspots may be a response to diverse environmental pressures acting on the genes residing in these hotspots. The maintained plasticity may then provide the mutational flexibility for these genes to adapt rapidly to changing selective pressures. Therefore, it is not surprising to see that multiple immune system-related genes are variable in copy number across primates, possibly resonating with the 'Red Queen hypothesis': that the constant diversification of the host immune system genes and the parasite defense genes is in response to changes in each other's defenses [21].

For example, we observed a significant enrichment of HCR CNVs in a chromosome 19 region corresponding to the leukocyte receptor cluster (LRC). In humans, this 1 Mb region encompasses several families of immunoglobulin (Ig)-like receptor genes, including gene clusters encoding multiple leukocyte Ig-like receptors (LILRs), leukocyte-associated Ig-like receptors (LAIRs) and killer-cell Ig-like receptors (KIRs). The KIRs have a multifaceted role in two processes, immune defense and reproduction, and interact with cell-surface molecules encoded by the MHC class I locus, another region that displays rapid evolution and copy number variation. These epistatic interactions likely require the co-evolution of MHC and KIR, similar to the co-evolution of parasitic and host defenses described above. Under ever-changing pathogenic pressures, more of this variation could be maintained, especially among primates, which, due to their complex social dynamics, have higher pathogenic transfer rates [22]. Therefore, at least some of these primate CNV hotspots are likely maintained under dynamic selective pressures, allowing for copy number variability at these loci.

Other gene ontological categories are represented, albeit less frequently, in the observed primate CNV hotspots. For instance, the pepsinogens (*PGA *family) are precursors for pepsin (a major digestive enzyme) and may be involved in local environmental adaptation of primates [23]. Such adaptation would be akin to that of the amylase encoding gene in humans, where different copy numbers of the amylase gene evolved as an adaptation to dietary habits [7]. Similarly, genes such as *CHYS1*, involved in wound healing, are also noteworthy. More surprising are gene families such as *PHDB *and *CBX*, which may be involved in neural function [24] and, among other functions, testis development [25], respectively. These findings provide an initial framework for functional studies to establish the extent to which the variation in these genes has contributed to primate evolution.

**Figure 5:**
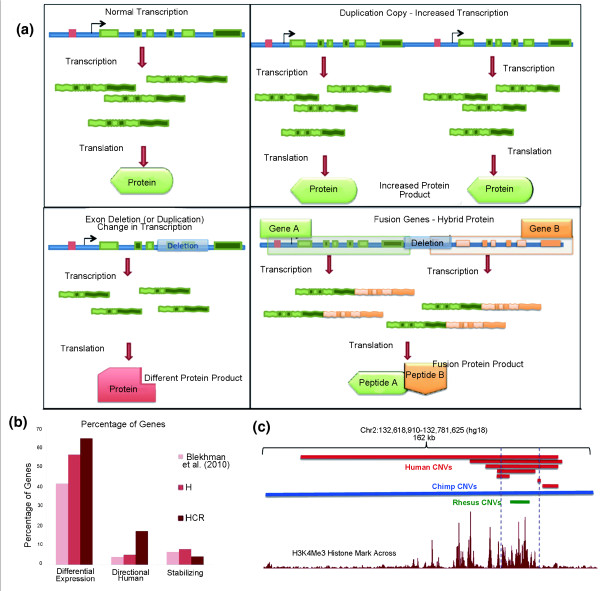
**Impact of CNVs on gene regulation**. **(a) **There are multiple ways in which CNVs can impact transcription by overlapping coding regions of the genes. **(b) **Blekhman et al. (2010) used RNA-seq data to determine whether specific genes are differentially expressed between human, chimpanzee, and macaque [32]. Based on their results, we plotted the proportion of human CNVs (H) and hotspot CNVs (HCR) that are differentially expressed between species. In particular, 3,423 of the Ensembl genes analyzed by Blekhman et al. (2010) overlap with human CNVs. Based on these data, here we plot the proportion of the genes that are differentially expressed between two or all three species, evolved under directional selection in the human lineage (Directional Human) or under stabilizing selection (that is, no expression differences between species). **(c) **At least three HCR CNVs overlap with regions with clear enhancer and/or promoter signals in the genome. To visualize the enhancer and promoter activity, we used the H3K4Me3 track generated by the ENCODE consortium [33] from the UCSC Genome Browser.

In their classic paper, King and Wilson [26] recognized the similarity between the macromolecules in chimpanzees and humans, noting that regulation of the amount of these macromolecules during different developmental phases may account for most of the phenotypic differences. In this theoretical framework, copy number variation may be one of the major mechanisms to regulate the expression levels within and between the species (Figure 5a). Indeed, genes that overlap with HCR CNVs were more likely to be differentially expressed between the three primate species studied here and to have evolved under positive selection in primates (Figures 4c and 5b). Further evidence indicates that intraspecific expression differences are also significantly higher in genes that fall into primate hotspots (Figure 5b; Figure S9 in Additional file 2). Not surprisingly, in addition to the HCR CNVs that overlap with coding regions of the genes, we found that at least two HCR CNVs overlap squarely with known enhancer regions that are highly conserved at the sequence level (Figure 5c). The redundancy in enhancers has been related to phenotypic robustness in fruit flies (*Drosophila melanogaster*), especially when exposed to genetic and environmental variability [27]. Hence, the maintenance of copy number variation in enhancer elements in primates may similarly reflect the evolutionary response to maintain phenotypic robustness in varying and rapidly changing selective pressures. By changing the number and position of genes or regulatory elements present in a single genome, CNVs likely impact gene regulation.

In addition, two recent studies demonstrated that copy number variation in one locus affects the expression levels in other loci. One of these studies showed that the expression level of a gene can be changed through alteration of the copy number variation of another gene that shares the same promoter region [28]. The other study demonstrated that the expressed pseudogene of *PTEN *acts as a sponge for microRNAs. As such, the deletion of the pseudogene subsequently increased the number of microRNA molecules, which can, in turn, negatively regulate the expression of the parental gene [29].

## Conclusions

This study provides a critical framework for describing and delineating the functional, biomedical and evolutionary impact of hotspots of CNV formation in primates. Our results underscore the significance of copy number variation as a widespread source of genomic variation among primates, and the implication of natural selection acting on these regions indicates that CNVs have contributed to the evolution of quantitative traits in primates.

## Materials and methods

The existing data for rhesus macaque CNVs [14] is limited. In order to produce a complementary CNV dataset, we identified and characterized CNVs among 17 unrelated rhesus macaques using a platform with a 15 kb effective resolution (Figure S2 in Additional file 2). Genomic DNA was obtained through the New England Primate Research Center (NEPRC) Primate Genetics Core. All animals in the study were housed at the NEPRC and maintained in accordance with the guidelines of the Committee on Animals of the Harvard Medical School and the Guide for Care and Use of Laboratory Animals of the Institute of Laboratory Animal Resources, National Research Council, Department of Health and Human Services, publication no. (NIH) 85-23, revised 1985. NEPRC is accredited by the American Association for the Accreditation of Laboratory Animal Care. All normalized Cy3/Cy5 intensity data from the aCGH experiments have been uploaded to the Gene Expression Omnibus (GEO) database under the accession number [GEO:GSE19881]. All CNV calls with corresponding log_2 _values are provided in Table S2 in Additional file 1.

We analyzed the patterns of these primate hotspots with respect to the human reference genome, due to the high level of accurate annotation of the human genome assembly compared to the draft chimpanzee and rhesus macaque reference sequences. We compiled a non-redundant human CNV dataset using data from two recent, high-resolution studies that had an effective resolution of 450 bp [1,15]. This dataset, which includes 12,146 CNVs, represents one of the highest resolution human CNV maps currently available. We have not incorporated recently released 1000 Genomes Project CNV calls because they are incomplete and biased towards deletions. As a chimpanzee CNV dataset, we used data primarily generated by Perry *et al. *[13].

To compare the locations of macaque and human CNVs, we used the Lift-Over tool developed by the UCSC Genome Bioinformatics Group to convert rhesus CNV loci to human coordinates (hg18). This computational tool utilizes the BLAT algorithm to align orthologous sequences between species [30]. Using this methodology, we were able to map 1,073 macaque CNVs (approximately 93%) onto the human reference genome. Chimpanzee CNVs were detected using an array design based on the human reference genome (hg18) and therefore subsequent conversion to human coordinates was not necessary.

For statistical calculations and visualization, we used the R statistical package.

## Abbreviations

aCGH: array comparative genomic hybridization; bp: base pair; CNV: copy number variant; HC: human chimpanzee hotspot; HR: human rhesus macaque hotspot; HCR: human chimpanzee macaque hotspot; kb: kilobase; KIR: killer-cell Ig-like receptor; Mb: megabase; MHC: major histocompatibility complex; NEPRC: New England Primate Research Center.

## Authors' contributions

OG and PLB carried out the experiments, conducted most of the preliminary analyses and planned the subsequent analyses. OG, PLB, RCI, QZ and XS conducted the population genetics analyses. REM designed the species-specific arrays. II conducted the frequency distribution analyses. EJW conducted initial selection analyses. AGC helped in planning the selection analyses and writing. OG, WEJ and CL conceived and wrote the study. All authors read and approved the final manuscript.

## Supplementary Material

Additional file 1 **Supporting tables**. The tables of the CNVs considered in the study and summaries of additional analyses. Click here for file

Additional file 2 **Supporting figures**. Supporting figures for the manuscript. Click here for file

Additional file 3 **Supporting methods**. Additional description of methodologies described in the manuscript. Click here for file
