# Supplementary material for: Refinement of primate copy number variation hotspots identifies candidate genomic regions evolving under positive selection
Source: Genome Biol. 2011 May 31;12(5):R52. doi: 10.1186/gb-2011-12-5-r52 (PMC3219974; doi:10.1186/gb-2011-12-5-r52)
Supplement: Additional file 2 — Supporting figures. Supporting figures for the manuscript. [file gb-2011-12-5-r52-S2.DOC]

**SUPPORTING FIGURE LEGENDS**

**Figure S1. Examples of array-based comparative genomic hybridization (aCGH) data.** **A)** To ensure the quality of the data, we ran two self-self experiments using the macaque sample that we used as the reference individual (354) in our hybridizations. Significant deviation of the log_2_ ratios from 0 indicate **B)** gains and **C)** losses, relative to the reference DNA.

**Figure S2. Inter-individual copy number variation among macaques.** **A)** The number of CNVs found in each macaque individual shows remarkable variation, from 25 to 330. **B)** To understand whether the observation of such genomic variance is an artifact or has biological significance, we plotted derivative log_2_ ratios (DLR, a measure of noise based on the distribution of log_2_ ratios, *X-axis*) with the number of CNVs called (*Y-axis*) and observed minimal correlation is minimal (R^2^ = 0.29). **C)** The ratio of common and singleton CNVs varies significantly and is independent of the number of CNVs observed in an individual (R^2^ = 0.004). These observations suggest that at least some of the observed variation of the number of calls made in each sample, relative to the reference, is not due to background noise, but due to differences in ancestral affinity of the samples to the reference sample. **D)** We also observed a significant correlation between chromosome size and the number of CNVs observed in respective chromosomes, consistent with the notion that the observed CNVs are randomly distributed across the genome (R^2^ = 0.853).

**Figure S3. The distribution of distances between probes across the macaque genome.** For this study, we custom-designed a platform containing 950,843 oligonucleotides from across the rhesus macaque genome. These probes are generally distributed uniformly across the genome, except for a handful of regions where a higher density of probes (~100 bp spacing) was used. As evident by the lack of a peak at that range, the small percentage of targeted probes did not change the overall distribution. We used asimilarity filter, which eliminated oligonucleotides that map to the reference genome more than once. Hence, the repetitive regions, including some segmental duplications and most of the centromeric regions are omitted as probe targets. The impact of this filtering is evident from the long tail of the distribution.

**Figure S4. Overview of the distribution of CNVs in the macaque genome.** **A)** The chromosomal distribution of CNVEs discovered in this study for the first 5 chromosomes (“Chr”). Green, red and blue vertical lines represent gains, losses and multiallelic CNVEs, respectively. The length of the line corresponds to the relative frequency. **B)** The number of CNVEs discovered in this study in comparison to the initial macaque CNV study - Lee et al. (2008) study [14]. **C)** The percentage of gains, losses and multiallelic CNVEs found in the present study. “Multi” refers to the multiallelic CNVEs, where both losses and gains relative to the reference were observed.

**Figure S5. Model-data comparison of CNVE frequency distribution**. Gamma-Poisson model was used to fit the observed frequency of CNVEs counts [34]. Specifically, we generated a model of frequency distribution in macaques based on our observations. This model uses a capture and recapture method to fit a Gamma-Passion model to the observed frequency distributions. Based on our model, we were able to estimate the lower-bound number of CNVs at the same size range that are yet to be seen among macaques, if further studies are conducted. Using this model, and arguments similar to Ionita-Laza et al. (2009), we estimated that analyses of 16, 32, 80 and 160 additional rhesus macaques would reveal at least 619, 1098, 2181 and 3490 new CNVs, respectively (Table S3).

**Figure S6. Enrichment analyses for macaque CNVs.** The plot depicted overlap analysis with 1,000 permutated macaque CNVE datasets (Mean and standard deviations are shown on each graph) comparable to **A)** Ensembl Genes, **B)** Segmental Duplications and **C)** Conrad et al. (2010) CNVEs. The x-axis is the number of overlapping CNVEs and the y-axis is the frequency of the permutated CNVEs. The dotted red vertical line indicates the observed number of overlaps.

**Figure S7. Size comparison of macaque CNVs that overlap and do not overlap with human CNVs.** 225 (20.97%) independent rhesus macaque CNVs overlap with human CNVs at least by 100 bp or more. The size distribution of the rhesus CNVs that overlap with human CNVs (light blue) are slightly larger than the 1160 macaque CNVs that we observed. However, they generally follow a similar size distribution and are not confined to a particular size class (i.e., no particular size bin in macaque CNVs that overlap with human CNVs is over-represented in relative to the distribution of the macaque CNVs in general).

**Figure S8. Hotspot regions are enriched for genes.** The plot depicts the overlap analysis of genes with 1,000 randomly-distributed permutated **A)** CNVs and **B)** hotspot regions (both mimicking the size distribution) with RefSeq genes. The red bars indicate the expected distribution and the dotted red line is the actual observation. This observation demonstrates that the enrichment of genes among HCR CNVs is not merely a bias from multiple CNVs that overlap the same genic region (e.g., multiple HCR CNVs overlap the HLA region) or from overlapping with UCSC gene track (rather than more conservative RefSeq gene track). **C)** The K values for all genes, genes that overlap with human CNVs, and genes that overlap with HCm HR and HCR CNVs. K is a measure of positive selection [18]. The genes that overlap with HCR CNVs have significantly lower K values (p<0.001, two-sample Kolmogorov-Smirnov test), indicating that they are more likely to evolve under positive selection. Note that the pattern is also evident in genes that overlap with HR CNVs, but largely lost in genes that overlap with HC CNVs. **D)** A cumulative fraction plot of conservation for primate hotspot and non-hotspot CNVs. Conservations scores were obtained using the phastCons on 17-species multiz track from the UCSC Genome Browser [35]. The D and P-values were calculated using a two-sample Kolmogorov-Smirnov test. Note the interesting trend that HR CNVs are more conserved than human CNVs. This observation, which may be due to enrichment of genic content among rhesus macaque CNVs, made the lack of conservation in HCR CNVs even more significant.

**Figure S9.** **Expression differences in HLA genes.** We plot the impact of a single event, CNVE2845 to the expression levels of several HLA genes. We used recently published RNAseq data for expression values and array based data for CNV genotypes [36]. In this figure, a single, large HCR CNV was substantially correlated with expression of three different genes.

**Figure S10.** **Overall expression differences.** We used recently released expression (“expn”) data from European individuals [37] to calculate a slightly modified z-score, which is a measure of the extent of variation normalized for comparison across different datasets (i.e., gene expression levels). This figure shows that the extent of expression differences is larger in genes that overlap with HCR CNVs. However, the result depends on only a handful of genes that overlap with HCR CNVs and are expressed in B-cells. Further, genome-wide studies focusing on the impact of CNVs to expression levels are warranted.

**Figure S11.** **The percentage of multi-allelic CNVs in all human CNVs and HCR CNVs.** Multi-allelic CNV defined here as a locus where a gain and a loss are observed across different individuals. However, since array-based approaches have difficulty in correcting for reference effects (e.g., if the reference has a single copy at a locus, diploid individuals would be called as a relative gain, respectively), these results should be interpreted with caution. The proportion of multi-allelic CNVs is slightly higher among HCR CNVs comparing to all observed human CNVs.

**Figure S12. Defining CNV regions and CNV elements.** We have defined CNV regions (CNVRs) as genetic loci that overlap with any clustered CNV calls. CNV elements (CNVEs) are defined within these CNVRs. If two CNV calls overlap more than 50%, they are merged into a single CNVE. If the reciprocal overlap is less than 50%, they were defined as different CNVEs.

**Figure S13**. **Reduced median networks of the common CNVs detected in macaques.** We generated binary reduced median (RM) networks from the common CNVEs of 17 macaque individuals using NETWORK v4.5.02 [38]. This software applies a parsimony-based algorithm to link individuals based on shared motifs (haplotypes) of phylogenetic characters. Using 328 common CNVs as unphased haplotypic characters, we coded each individual macaque by the absence (0) or presence (1) of each character, and ran the RM calculations on the resulting 328 by 17 binary matrix. Red circles represents CNV coded-haplotypes that are not seen in any samples. The gray circles indicate coded-haplotypes of samples, which are designated by the numbers inside the circles.

**Figure S1**

**Figure S2.**

**
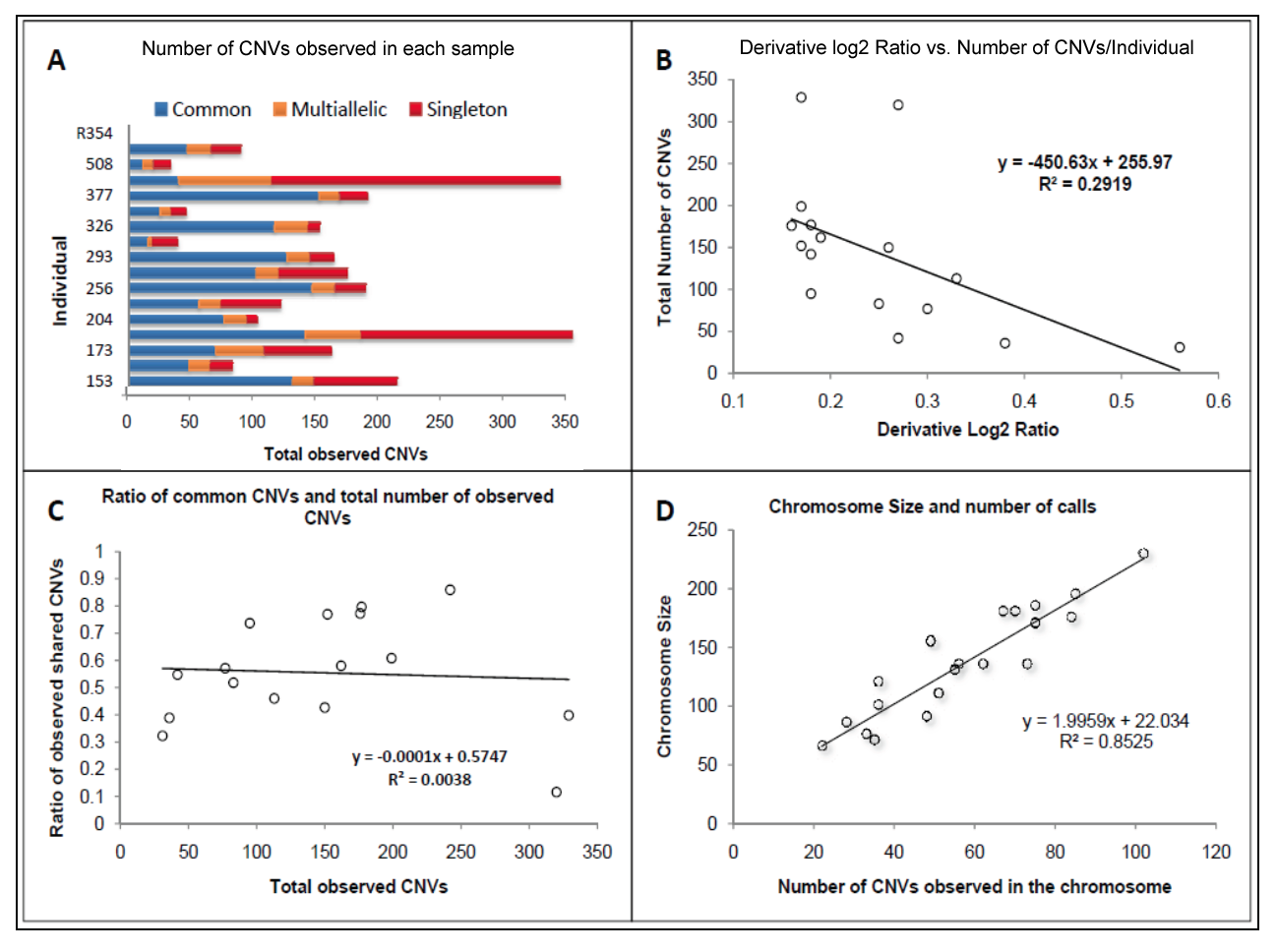
**

**Figure S3.**

**
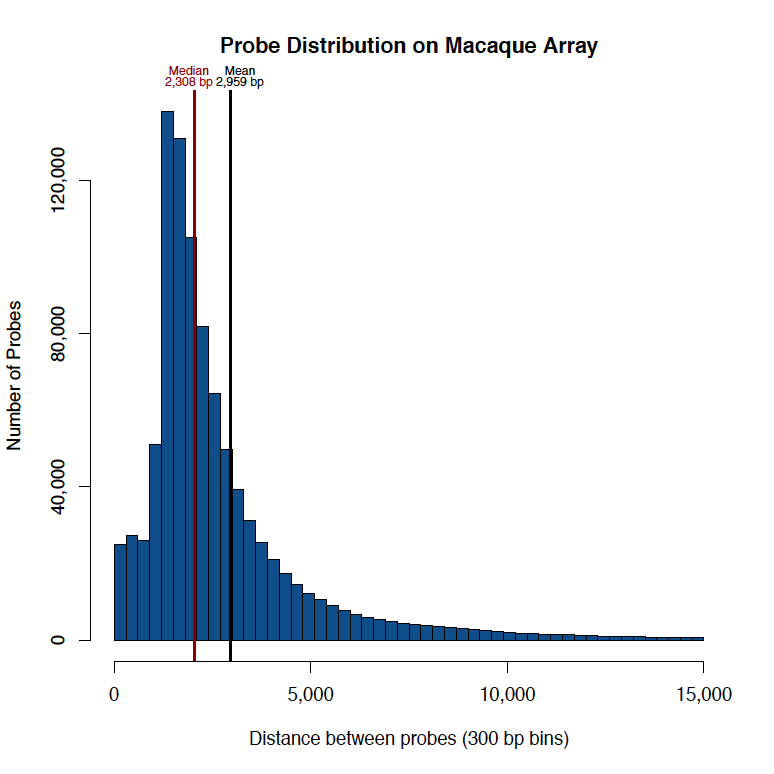
**

**Figure S4**


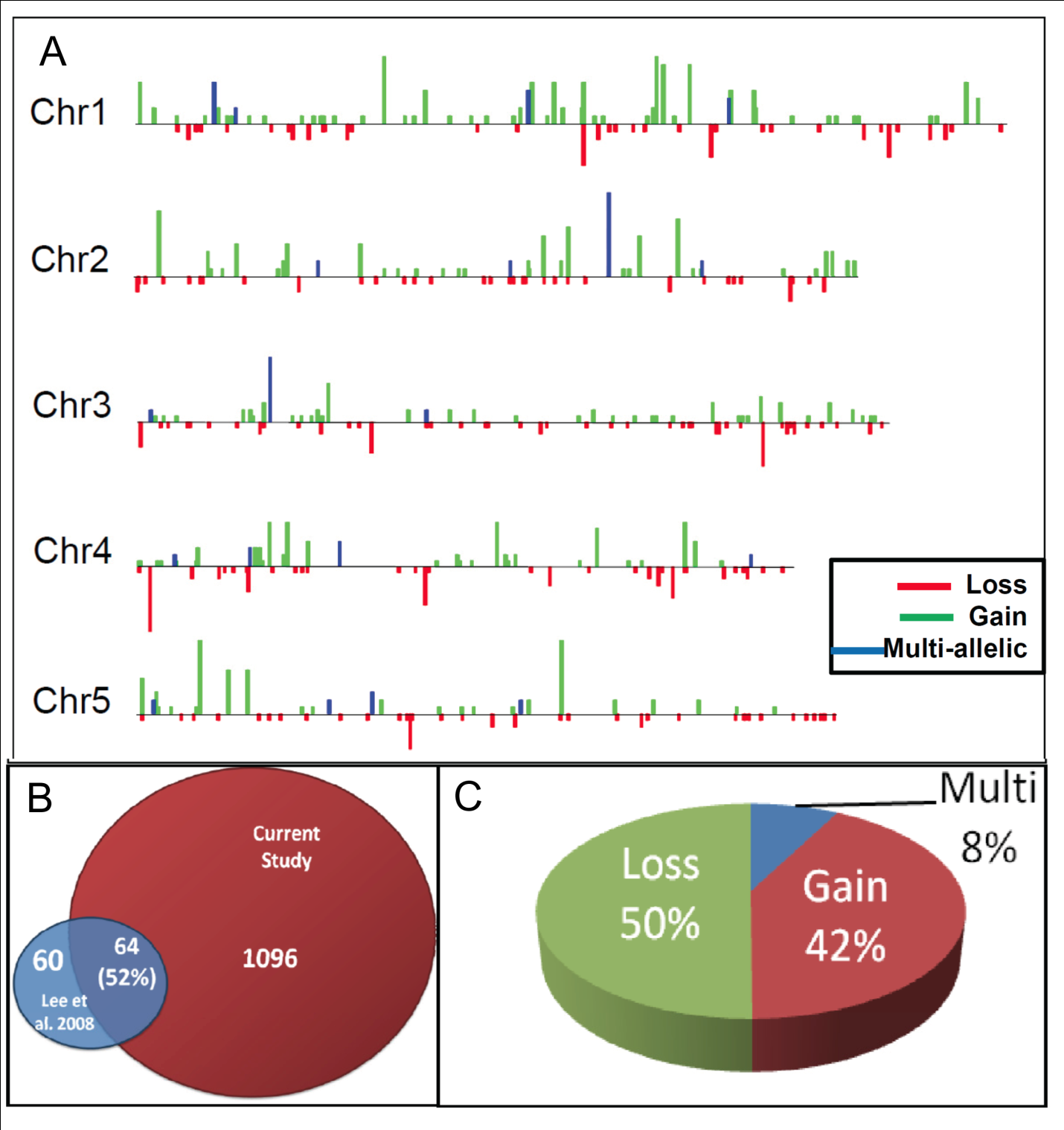


**Figure S5**

**
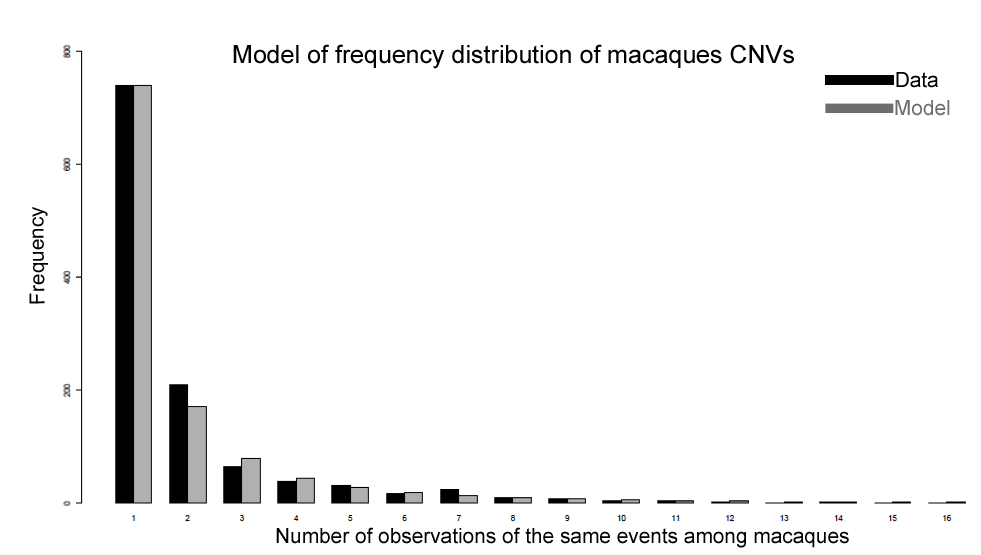
**

**Figure S6.**

**
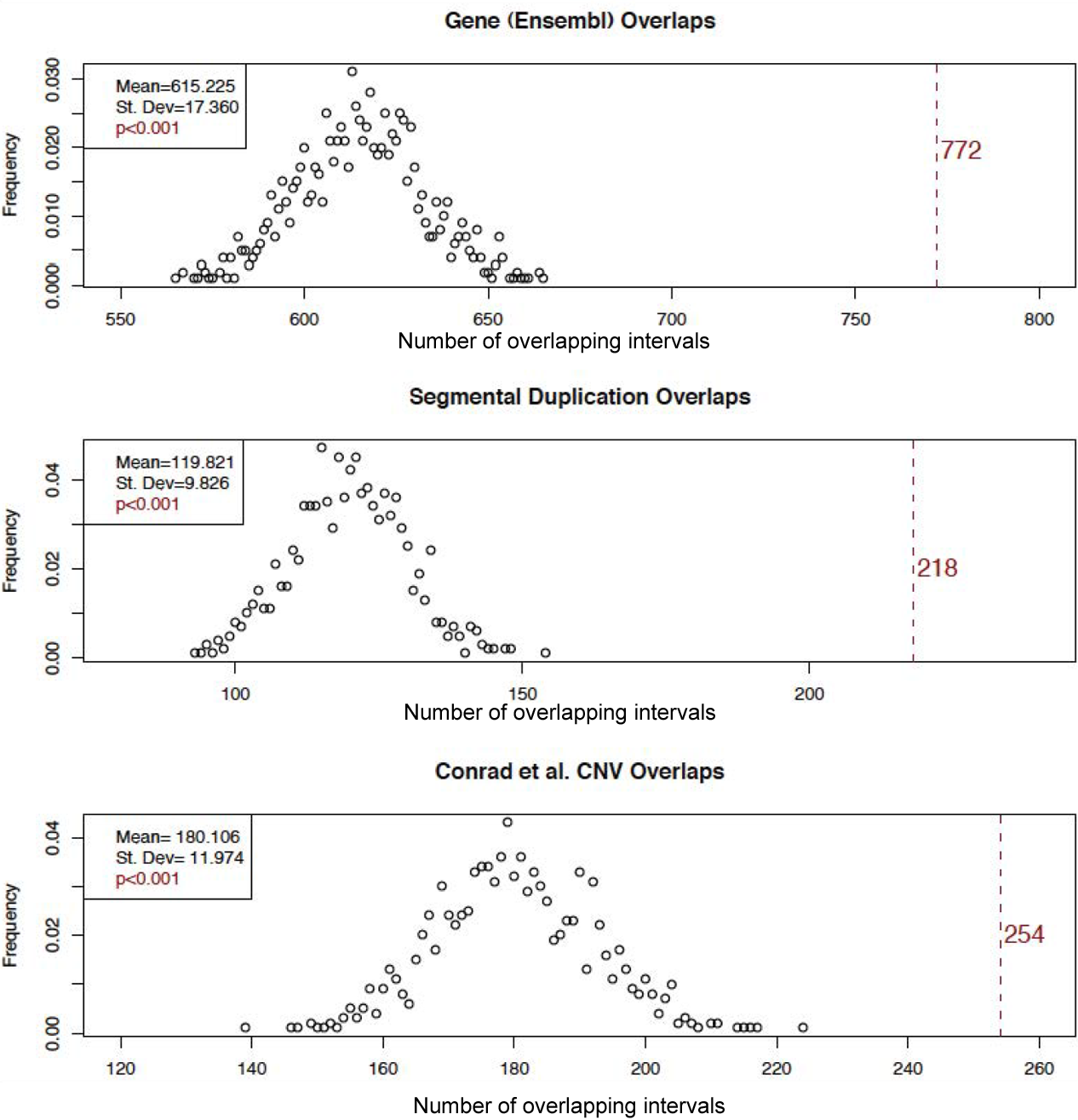
**

**Figure S7.**

**
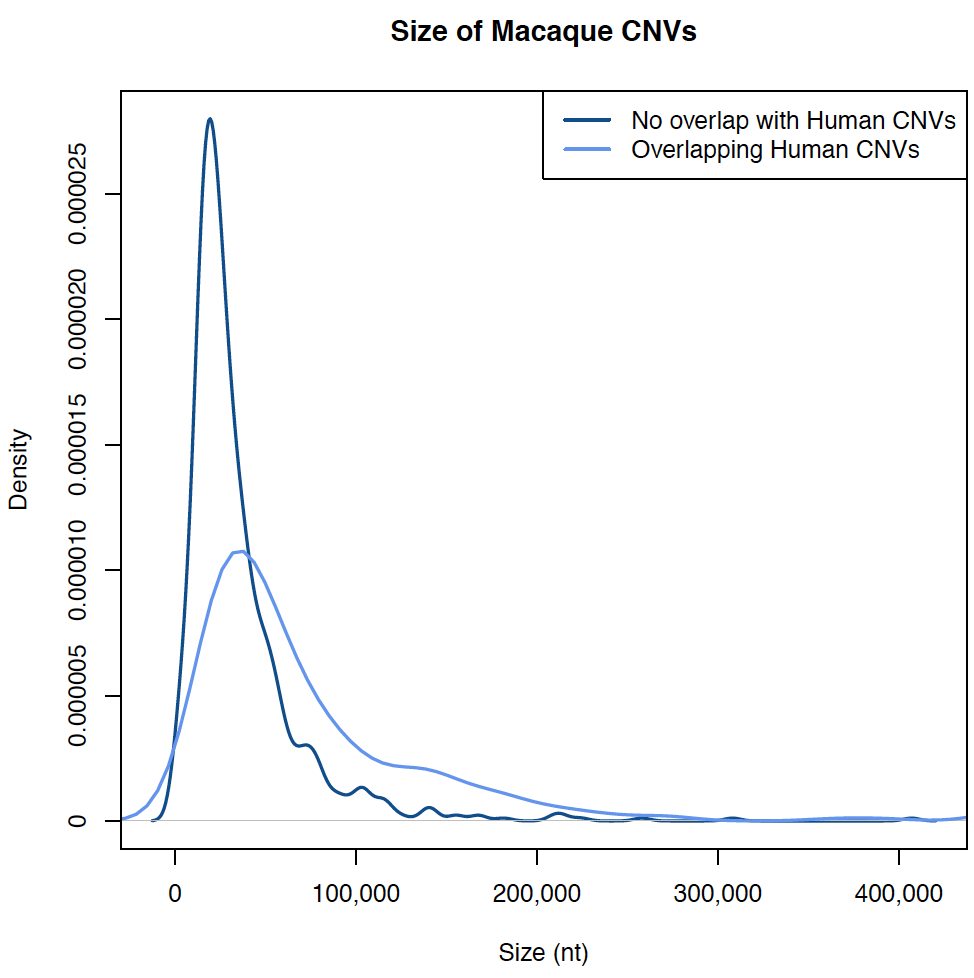
**

**Figure S8.**

**
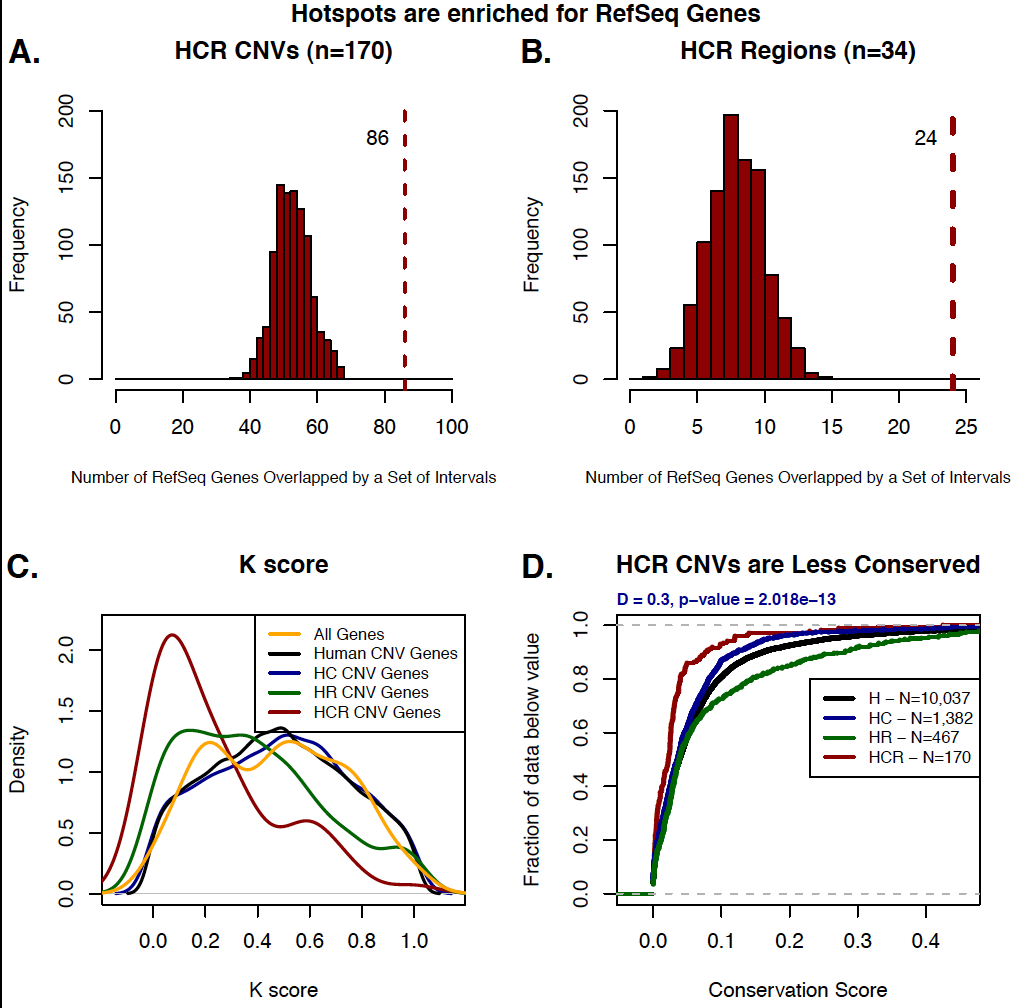
**

**Figure S9.**

**
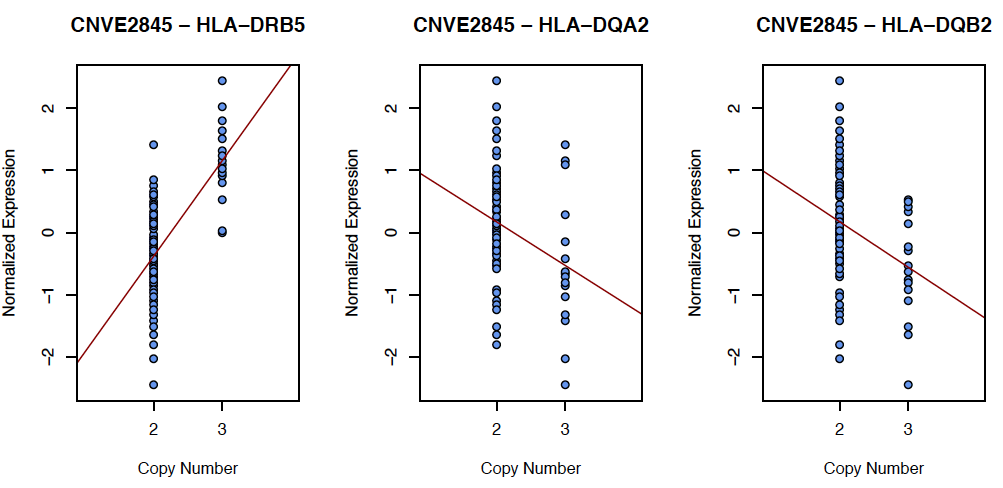
**

**Figure S10.**

**
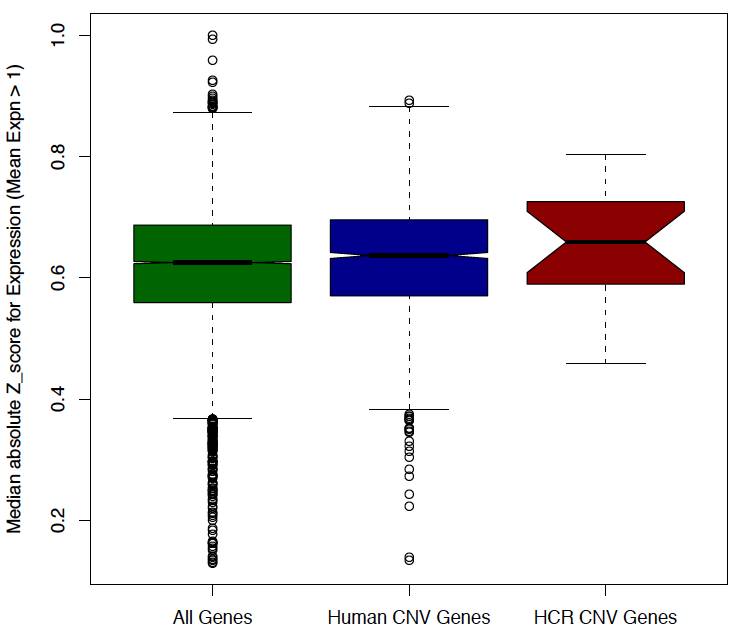
**

**Figure S11.**

**
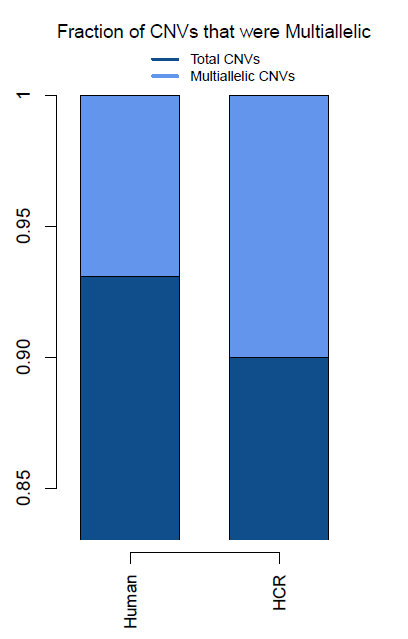
**

**Figure S12.**

**Figure S13.**

**
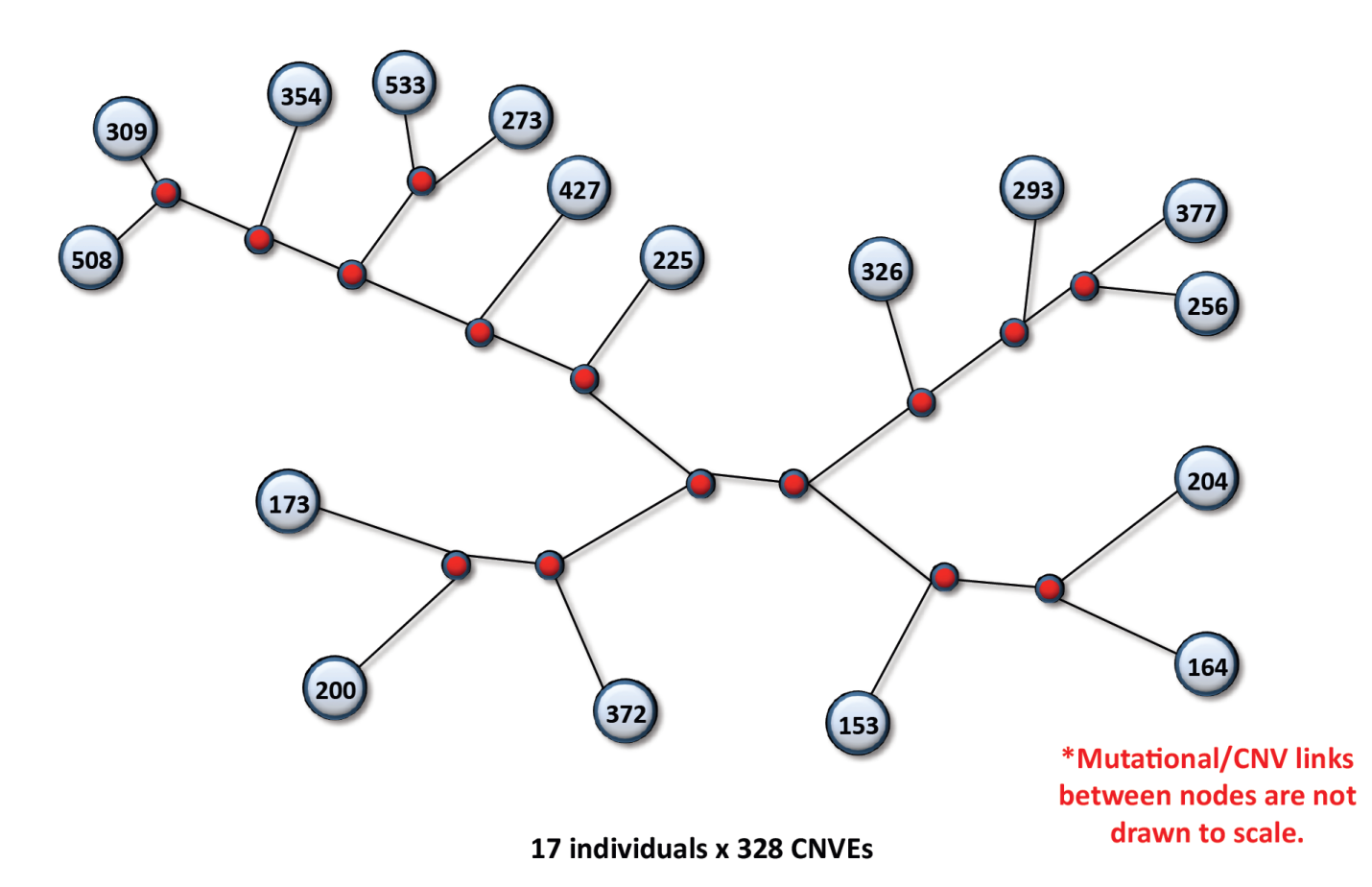
**
