# Supplementary material for: Refinement of primate copy number variation hotspots identifies candidate genomic regions evolving under positive selection
Source: Genome Biol. 2011 May 31;12(5):R52. doi: 10.1186/gb-2011-12-5-r52 (PMC3219974; doi:10.1186/gb-2011-12-5-r52)
Supplement: Additional file 3 — Supporting methods. Additional description of methodologies described in the manuscript. [file gb-2011-12-5-r52-S3.DOC]

**SUPPORTING METHODS**

**Sample collection and preparation**

Rhesus macaques (*Macaca mulatta)* are old world monkeys that belong to the Family *Cercopithecidae* and share a common ancestor with great apes that existed about 25 million years ago [39]. As such, they are an ideal phylogenetic outgroup for understanding the evolution of great apes (Hominoidea). Furthermore, macaques are second only to *Homo sapiens* in terms of geographical range and ecological diversity, with representatives found as far apart as the African Barbary coast (*M. sylvanus*) and Japan (*M. fuscata*). In the past, macaques occupied an even broader range, with fossil specimens found throughout Europe, Africa and Asia. As a result of this rich phylogeographic history, their close evolutionary relationship to humans, and the endangered status of several species, macaques are a major focus of anthropological research. In addition, Asian macaques (most notably rhesus macaques from India and China) are widely used as experimental models of human behavior and disease, particularly in scientific areas where small animal models are either unavailable or insufficiently similar to humans (e.g., behavioral biology, reproductive biology, and infectious diseases). Indeed, the rhesus macaque is now the most widely used animal model for research related to HIV/AIDS pathogenesis [e.g., 40, 41].

Genomic DNA was obtained through the New England Primate Research Center (NEPRC) Primate Genetics Core. All animals in the study were housed at the NEPRC and maintained in accordance with the guidelines of the Committee on Animals of the Harvard Medical School and the Guide for Care and Use of Laboratory Animals of the Institute of Laboratory Animal Resources, National Research Council, Department of Health and Human Services, publication no. (NIH) 85–23, revised 1985. NEPRC is accredited by the American Association for the Accreditation of Laboratory Animal Care. Six milliliters of venous blood was collected in EDTA-Vacutainer tubes during routine physical exams in which blood testing was already ongoing. Genomic DNA was then isolated and purified using Flexigene kits (Qiagen, Valencia, CA) following the manufacturer’s standard protocols.

**Array design**

In this study, we used a custom designed Agilent array-based comparative genomic hybridization (aCGH) platform to compare the genomic DNAs of 16 unrelated rhesus macaques of Indian origin to the genome of an unrelated male sample (R354) from the same species. The platform comprises 950,843 unique 60-mer oligonucleotide probes specific to the rhesus macaque reference genome (rheMac2). The remaining 30,000 spots on the platform were used for positive and negative controls (6,685 probes), 1,000 probes replicated 5 times throughout the array (5,000 total), and 11,488 normalization probes. The first two sets of spots allow quality control measures, whereas the normalization probes allow between-array normalizations, as necessary. The probes are annotated with macaque chromosomal locations based on the reference genome (rheMac2) [39], and are randomly distributed on each chip. It is important to note that probes designed on the basis of the human reference genome yield very poor results when hybridized with macaque samples [42]. Fourteen genic regions, comprising approximately 400 kb of genomic content (Table S4) were enriched for probes and interrogated at higher resolution than rest of the genome. This enrichment has not significantly changed the overall resolution of the array since only <0.3% of the probes fall into these enriched regions.

To minimize false-positives and the misannotation of CNV calls, we applied a strict similarity filter on the oligonucleotide probes that are used to construct the array platform. This filter eliminates probes that perfectly map to the macaque reference genome more than once, leaving only the probes that align to a unique location in the reference macaque genome (rheMac2). Hence, simple repeats, most of the known segmental duplications and retrotransposed elements are effectively avoided on this platform. In addition, it should be noted that more than ~8% of the draft macaque genome consists of sequence gaps [39] that the aCGH platform in this study was not able to interrogate.

**Array-based comparative genomic hybridization**

Sixteen individual rhesus macaque genomes were compared to a reference macaque genome (R354) on custom-designed 1M oligonucleotide-based (Agilent Technologies) aCGH slides per manufacturer’s recommendations. Each sample was diluted to 1.0 µg genomic DNA per 21 µL of sterilized water in 1.5 mL microcentrifuge tubes and fragmented via water-bath sonication (SONICS, Vibra-Cell) for 1 minute, using 100 amplitude pulses in 10 second intervals. Test DNAs were labeled with Cy5 and co-hybridized with Cy3-labeled DNA from the reference individual (R354).

All experiments were labeled and hybridized using standard Agilent aCGH protocols. DNA labeling was performed with Cy3-dCTP or Cy5-dCTP (Invitrogen) using Exo-Klenow DNA polymerase (New England Biolabs) at 37°C for 2 hours, and labeled products were purified through YM-30 microcentrifuge filter columns (Millipore). Labeled test and reference DNAs were then combined with 50µg human Cot-1 DNA (Invitrogen) and the appropriate buffering reagents, applied to the aCGH microarray slide, and hybridized at 65°C for 40 hours in rotisserie incubators at 20 rpm. Arrays were subjected to two rounds of post-hybridization washing, and then scanned with a 2 micron resolution Agilent microarray scanner (Agilent Technologies). Signal intensities and array coordinates were extracted using FeatureExtraction v10.7 software (Agilent Technologies), and derivative log_2_ ratios were assessed for run quality and batchconsistency. Extracted data files were then imported into Nexus 4 (Biodiscovery, Inc.) for CNV analyses. All normalized Cy3/Cy5 intensity data from the aCGH experiments have been uploaded to the Gene Expression Omnibus (GEO) database under the accession number GSE19881. All CNV calls with corresponding log_2_ values are provided in Additional File 1: Table S1.

**Identifying CNVs**

To obtain a false-positive rate, we conducted two self-self experiments using the reference sample as both reference and test. These runs were consistent and gave less than 5 aberrations with an absolute log_2_ ratio of 0.35 or greater. In self-self experiments we expect to have no significant aberrations and the ones that are detected indicate possible false-positives. Hence, we removed the calls that overlap with the aberrations that were spotted in both of the self-self experiments. We used NEXUS 4 (Biodiscovery, Inc.) to identify CNVs, and visually identified each CNV call (Figure S1). Finally, to minimize false positives, we filtered out singleton CNVs (i.e., those do not have 50% reciprocal with any other CNV) that had average log_2_ ratios between 0.35 and -0.35. Using this process, we identified 2,284 CNVs among 17 unrelated rhesus macaques. To finalize the dataset, we followed a two-step procedure (Figure S12). First, we identified CNV regions (CNVRs), defined as clusters of CNVs with at least a 1 bp overlap. Second, within these CNVRs, we identified CNV elements (CNVEs), defined as segments comprised of two (or more) CNVs with at least 50% reciprocal overlap. Overall, we discovered 1,160 CNVEs across 1084 CNV regions. The cumulative genomic content of these CNVEs per macaque, corresponds to 58.1 Mb of sequence, covering slightly more than 1.9% of the rhesus macaque genome.

**Validation**

To validate the aCGH results, we randomly selected 40 common CNVEs and were able to independently validate 31 of these regions using quantitative polymerase chain reaction (qPCR) assays, using at least 2 individuals for each CNVE. The remaining 9 tests were inconclusive (i.e., the standard deviation of the qPCR results was too high for conclusive validation). Six singletons were also validated and all 6 qPCR amplifications were consistent with the array results. We detected 64 of the 124 (~52%) CNVEs that were initially reported in Lee et al. [14]. 743 of the 1,160 (~64%) CNVEs documented in this study were singletons (i.e., the variants that are detected in a single individual, Figures S2A-S2C). This is concordant with previous observations of singletons among humans (49% among 40 individuals) [1], chimpanzees (76% among 30 individuals) [2] and macaques (76% among 9 individuals) [14], indicating similar frequency spectra for CNVs across old world primates. Similar to human CNVEs, macaque CNVEs are comprised predominantly of losses (~50%), followed by gains (~42%), and multiallelic CNVEs (~8%), relative to the reference individual. The number of CNVEs detected on each chromosome is correlated with the size of the chromosomes (R^2^= 0.8525, Figure S2D), indicating that the CNVEs are more or less uniformly distributed across the chromosomes.

**Frequency distribution estimations**

It is reasonable to predict that there are thousands of common, smaller CNVs that have yet to be discovered among macaques. Hence, based on our results, we formally estimated the lower bound frequency of unseen CNVEs in the rhesus genome that would be detected if larger sample sizes were analyzed. To do this, we employed a capture and recapture method as described in Ionita-Laza et al. [34] to construct a Gamma-Poisson model corresponding to the CNV call set. To formally estimate a lower bound on the number of unseen CNVs in the rhesus genome, we employed a capture-recapture method [43] and fitted a Gamma-Poisson model to the corresponding pre-validated CNVE call data. We assume that at each CNV location, a CNV event occurs according to a Poisson process with mean lambda, where lambda is distributed as a Gamma with parameters (alpha, beta). The parameters alpha and beta of the Gamma distribution are estimated using maximum likelihood estimation from the data available on 16 individuals. We note here that the fit of this model to the data available on 16 macaque individuals is very good (Figure S5). Using this Gamma-Poisson model, and arguments similar to lonita-Laza et al. (2009), we estimated that analyses of 16, 32, 80 and 160 additional rhesus macaques would reveal at least 619, 1098, 2181 and 3490 new CNVs, respectively (Table S3).
